# The Effect of Endotoxin-Induced Inflammation on the Activity of the Somatotropic Axis in Sheep

**DOI:** 10.1155/2024/1057299

**Published:** 2024-08-07

**Authors:** Maciej Wójcik, Dorota Anna Zieba, Joanna Bochenek, Agata Krawczyńska, Marcin Barszcz, Alina Gajewska, Hanna Antushevich, Andrzej Przemysław Herman

**Affiliations:** ^1^ The Kielanowski Institute of Animal Physiology and Nutrition Polish Academy of Sciences, Instytucka 3 Street, Jabłonna 05-110, Poland; ^2^ Department of Nutrition and Animal Biotechnology, and Fisheries Faculty of Animal Sciences University of Agriculture in Krakow, Krakow 31-120, Poland

## Abstract

The hypothalamic-pituitary-somatotropic (HPS) axis controls many physiological and pathophysiological processes. The phenomenon of insensitivity to growth hormone resistance (GHres) was previously reported to be due to the development of inflammation. Therefore, the primary aim of the study was to determine the impact of inflammation caused by lipopolysaccharides (LPS) on the secretory activity of the HPS axis in sheep. The further goal was to determine the effect of inflammatory factors on individual components involved in intracellular signal transduction to GH via the GH receptor (GHR). The research was carried out on 24 seasonal sheep kept under a short-day photoperiod, randomly divided into two groups. Before the experiment, the sheep estrous cycles were synchronized. The results of the current study in a sheep model showed that inflammation impairs the activity of the somatotropic axis. On the one hand, LPS injection stimulated (*p* < 0.01) GH secretion, and on the other hand, it reduced the liver's sensitivity to this hormone by directly reducing (*p* < 0.01) GHR expression and activating the GHR inhibitory signal transduction mechanism. A symptom of such an inhibitory postreceptor signaling pathway may be due to an increase in SOCS3 expression (*p* < 0.01). The effect of various inhibition pathways is a significant reduction in the expression of the main transcription activator IGF1-STAT5B (*p* < 0.05). The action of GHres in the liver resulted in the inhibition of IGF1 secretion, which in the long term may have negative consequences for growth and development. Our study suggests that disruption of the GH cell signaling pathway may be one of the important elements of the pathophysiology of inflammation. It can suppress growth and hepatic metabolism to spare energy expenditure.

## 1. Introduction

The inflammation disrupts the homeostasis of an organism and leads to endocrine system disorders [1–3]. To induce systemic inflammation, experimental animals are treated with a lipopolysaccharide (LPS), which is a part of the outer cell membrane of Gram-negative (−) bacteria. The inflammation mediators, which exhibit pleiotropic activity, are believed to play a crucial role in the communication between immunological and neuroendocrine systems [4]. One of the endocrine axes, in which activity is modulated during inflammation, is the hypothalamic-pituitary-somatotropic (HPS) axis. Resulting of the LPS treatment systemic inflammation induces changes in the secretory activity of three major organs related to the HPS axis, namely, the hypothalamus, pituitary, and liver [1–3]. The hypothalamus is the main regulatory organ in this axis. Secreted by its nuclei neurohormones, growth hormone-releasing hormone (GHRH), which is secreted mainly in the arcuate nucleus (ARC), and somatostatin (SST), which is secreted mainly in the periventricular nucleus (PVN), are two main factors that affect the secretion of growth hormone (GH) in the anterior part of the pituitary (AP). The GH secreted from the pituitary gland is bound by the growth hormone-binding protein (GHBP). The complex thus created is finally able to bind with the GH receptor (GHR) [5]. GHR expression was observed in most tissues and organs, but it is mainly secreted in the liver, where its activation by the GH complex bound stimulates the insulin-like growth factor 1 (IGF1). It is believed that inflammatory mediators directly influence the synthesis and/or release of GH at the pituitary level, omitting the hypothalamic regulation of GH secretion. In addition, it has been observed that activation of the immune system inhibits the secretion of IGF1 [6]. However, this effect seems to be attributed to the decrease in liver sensitivity to the GH action caused by inflammation [7]. It is worth noting that the increase in GH secretion is a common element of the pathophysiology of sepsis both in sheep and primates including humans [7, 8], which suggests that sheep, as opposed to rodents, can be useful model animals in the studies considering the effects of interaction between the immune system and somatotropic axis. Therefore, the results of the studies on sheep can be valuable and useful for human medicine.

In some pathophysiological conditions, the mammalian organism was found to not respond to the action of GH commonly despite the high circulating level of this hormone. This specific body reaction is known as growth hormone resistance (GHres). The GHres symptoms seem to be homologous to those in the GH deficiency. GH deficiency may cause changes in the composition of the body, causing a reduction in lean body mass and an increase in fat mass [9], a reduction of the total amount of water in the organism [10], a decrease in bone density [11], a decrease of strength and endurance of muscles [12], harmful influence on the cardiovascular system [13], decrease in resting energy expenditure [14], decrease in protein metabolism and synthesis [15], carbohydrate metabolism disorders [15], lipid and lipoprotein metabolism disorders [16, 17], reduction of the total amount of collagen in the skin [17], changes in the organisms immune system response [15], and breeding disorders that manifest as a negative influence on, e.g., ovarian follicle development and puberty [18]. Causes of the GHres can be divided into the following two groups: inborn and acquired. Inborn GHres is, otherwise, known as Laron syndrome and it is caused by a genetic mutation of GHR. On the other hand, the acquired GHres was found to be caused by numerous factors such as inhibiting antibodies, malnutrition, diabetes, and renal or hepatic disorders [19]. An increasing number of experimental data indicates that one of the factors that induce acquired GHres may be inflammation. Most authors indicate the potential role of fibroblast growth factor 21 (FGF21), a protein that is as of yet poorly studied, in the induction of GHres. FGF21 is a protein hormone that regulates the adaptation of organisms to various conditions such as limited nutrients, cold, the amount of carbohydrates in diet, or stress, including immune stress. This factor can potentially influence the target tissues through 4 fibrogenic growth factor receptors (FGFR1–4). FGF21 affects the entire organism and acts in an endo- or autocrine fashion, depending on the stimulus and the production sites [20]. FGF21 is mainly synthesized in the liver [21] and adipocytes [22]. It was found that in humans, the level of FGF21 is positively correlated with the Quetelet II indicator also known as the body mass index or BMI and GH level but negatively correlated with the IGF1 level [23]. Moreover, its inhibiting influence on the activity of the signal transducer and activator of transcription (STAT)5b in the liver [24] which with Janus kinase 2 (JAK2) is part of the main pathway through which GHR transduces the GH signal [25, 26]. These results suggest the potential role of FGF21 in GHres induction. The stimulation of the immune system with LPS caused the development of the GHres as well [6]. FGF21 can potentially mediate in the GHres mechanism, as its level increases after LPS administration, inhibiting the expression of GHR-STAT5B. Also, the influence of LPS on JAK2 and STAT5B seems to be a confirmation of this thesis [27]. Moreover, it was found that FGF21 may be involved in the modulation of the inflammatory response due to its role in inhibiting the secretion of proinflammatory cytokines such as IL-1*β*, IL6, and TNFA [28]. The abovementioned facts appear to indicate that FGF21 induces GHres independently of proinflammatory cytokines and the only role of immunological stress is to activate FGF21. It is suggested that SIRT1 is involved in the regulation of FGF21 expression. However, this relationship was studied mainly in the context of metabolism regulation in the peripheral tissues such as the liver [29] or heart [30]. Sirtuin 1 (SIRT1) is a member of the sirtuins family (SIRT1–7), which are highly conserved NAD + - dependent protein deacetylases and/or ADP ribosyltransferases [31]. Sirtuins are considered to be one of the crucial regulators of a variety of cellular processes, including energy metabolism, stress response, tumorigenesis, and aging [32]. In turn, in adipocytes, the SIRT1⟶FGF21 pathway seems to be reversed as Chau et al. observed that it was FGF21 that regulated SIRT1 expression [33]. Also, the inhibiting influence of SIRT1 on the GH-induced GHR phosphorylation of STAT5B at the level of the liver [34], which is the main mediator of growth hormone signal transduction among STAT proteins phosphorylated by GHR [35]. Besides metabolism regulation, the role of SIRT1 was observed also in the modulation of the immune response. Liu et al. (2013) suggested that TLR4 signaling recruits SIRT1, which is then associated with the autoinhibitory mechanism of inflammation [36].

Considering the unknown mechanism of inflammation-induced resistance to growth hormone, the study's primary aim was to determine the influence of the inflammation on GH concentration and GH receptor expression, as well as the protein expression of GHR and IGF1 in the liver in the pathological state. Furthermore, genes' expression involved in the GH signaling pathway was determined on the level of the hypothalamus, anterior pituitary, and liver.

## 2. Materials and Methods

### 2.1. Animals

The *in vivo* experiment was conducted on 24 adult (2 years old, average weight was 58 ± 3 kg) blackface ewes in November. The ewes were kept indoors in individual pens and exposed to natural daylight. To mitigate the stress of social isolation, the animals had visual contact with each other. The ewes were fed a consistent diet of commercial concentrates, with hay and water available ad libitum. Twelve hours before the experiment began, the animals were deprived of food. Prior to the experiment, the estrous cycle stages of the ewes were synchronized using the Chronogest® CR (Merck Animal Health, Boxmeer, the Netherlands), following the methodology outlined in Przybył et al. [37]. The experiment was performed on the 10th day of the luteal phase, coinciding with the plateau levels of estradiol and progesterone. A venous catheter was inserted into the jugular vein the day prior to the experiment. Twenty-four ewes were randomly divided into the following two time-dependent groups: 3 hours (*n* = 12) and 9 hours (*n* = 12). Each time group was further divided into the following two subgroups: control (saline-treated; *n* = 6) and LPS treated (400 ng/kg body weight LPS from Escherichia coli, Sigma-Aldrich, St. Louis, MO, USA; intravenous (i.v.); *n* = 6).

Animals were euthanized 3 h or 9 h after i.v. injection of LPS, respectively. Tissues from the mediobasal hypothalamus (MBH) containing the arcuate nucleus (ARC), dorsomedial hypothalamus (DMH) containing periventricular nucleus (PVN), anterior pituitary (AP), and liver were collected and immediately frozen in liquid nitrogen and stored at −80°C until further analysis. Blood samples were collected every 15 minutes beginning 2 hours prior to the administration of LPS or saline, according to whether the group was LPS treated or served as the control.

Experimental procedures were approved (authorization no. WAW2/052/2018 from 23 March 2018) by the 2nd Local Ethics Committee of the Warsaw University of Life Sciences–SGGW (Warsaw, Poland).

### 2.2. qRT-PCR Assay

The gene expression analysis was performed according to the previously described protocol [37], with the use of NucleoSpin RNA/Protein kit (Macherey-Nagel, Dueren, Germany) to isolate the total RNA from collected tissues, Maxima™ First Strand cDNA Synthesis Kit (Thermo Fisher Scientific, Waltham, MA, USA) for a reverse transcription, and FIREPol® HOT EvaGreen qPCR Mix® Plus kit (Solis Biodyne, Tartu, Estonia) for real-time PCR. The primers used for each gene are listed in Table 1. PCR reactions were performed using a Rotor-Gene Q thermocycler (Qiagen, Germantown, MD, USA) with Rotor Gene Q software. The NormFinder (Molecular Diagnostic Laboratory, Arhus University Hospital, Arhus, Denmark) was used to identify the optimal normalization gene, from among the selected genes: glyceraldehyde 3-phosphate dehydrogenase (GAPDH), histone deacetylase 1 (HDAC1), beta-2-microglobulin (B2M), and cyclophilin C (PPIC).

The results are presented in arbitrary units, calculated as the ratio of the target gene expression to the expression of the reference gene, with the appropriate control group normalized to a value of 1.

### 2.3. Radioimmunological Assay

The concentration of GH in plasma was determined using a double-antibody radioimmunoassay (RIA) method, employing antibovine GH and anti-rabbit *γ*-globulin antisera, along with a bovine GH standard (NIDDK-GH-B-1003A). The detailed characteristics of the antiserum and the assay method were thoroughly described by Dvorak et al. [42]. The assay's sensitivity for GH was 0.6 ng/mL, with intraassay and interassay coefficients of variation at 5.9% and 10.2%, respectively.

### 2.4. ELISA

Tissue samples were homogenized in 1 ml of ice-cold phosphate-buffered saline (pH 7.4) and then subjected to two freeze-thaw cycles to further disrupt the cell membranes. Then, the samples were centrifuged for 5 min at 10000 × *g* and 4°C. The supernatants were collected and stored at −80°C for further analysis. The concentrations of GHR and IGF1, IGF1R, and STAT5B were determined using the following ELISA kits according to the manufacturer's instructions: Sheep GHR (growth hormone receptor) ELISA Kit, Sheep Insulin-like growth factor 1 ELISA Kit (ELK Biotechnology CO., Ltd., Denver, CO, USA). Total protein concentration was determined spectrophotometrically using the Bradford method and the Bio-Rad Protein Assay Kit II (Bio-Rad, Hercules, CA, USA). All absorbance measurements were performed on a SpectraMax iD3 microplate reader (Molecular Devices, San Jose, CA, USA).

### 2.5. Statistical Analysis

The statistical analysis was performed using TIBCO Statistica 13.3 (TIBCO Statistica Ltd., Palo Alto, CA, USA). The significant differences in proteins and gene expression between the experimental groups were determined using the Student's *t*-test to compare expression between the control and LPS-treated groups. Similarly, the concentration of GH in serum was determined using the Student`s *t*-test, and the last two measurements before slaughter were taken into account for the calculations. The results are presented as the mean ± standard error of mean (SEM) and results *p* ≤ 0.05 were deemed statistically significant.

## 3. Results

### 3.1. Effect of Peripheral LPS Injection on the Circulating Concentration of GH

**Figure 1:**
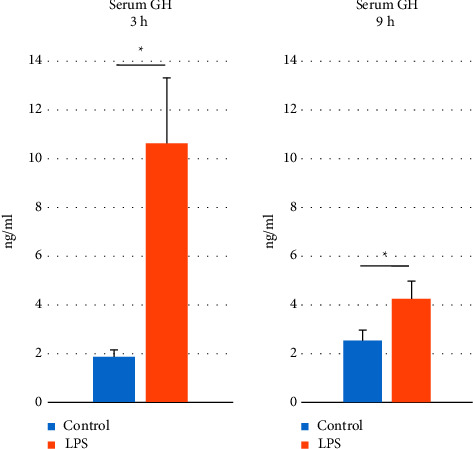
The effect of lipopolysaccharide (LPS; 400 ng/kg; iv.) injection on the serum growth hormone (GH) concentration which was measured 3 and 9 hours (h) after the treatment. Significant differences were analyzed by the Student's *t*-test. The results are presented as the mean ± standard error of mean (SEM) and results *p* ≤ 0.05 were deemed statistically significant. Asterisk (*∗*) shows the statistically significant differences between controls and research groups.

It was found that the plasma concentration of GH was increased (*p* < 0.05) in the LPS-treated groups both 3 and 9 h after the treatment in comparison to controls (Figure 1).

### 3.2. Effect of Peripheral LPS Injection on the Protein Expression of GHR and IGF1 in the Liver

**Figure 2:**
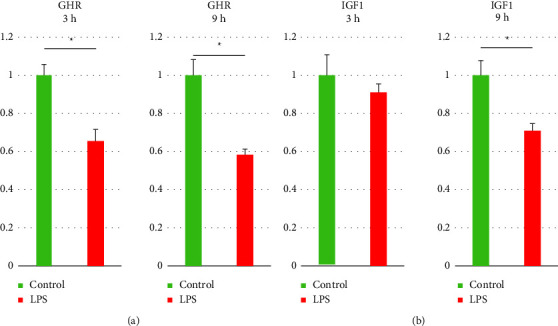
The effect of lipopolysaccharide treatment on the liver level of growth hormone receptor (GHR) (a) and insulin-like growth factor 1 (IGF1) (b) protein expression 3 and 9 hours (h) after the treatment. Statistically significant differences were analyzed by the Student's *t*-test. The results are presented in arbitrary units as the mean ± standard error of the mean (SEM) and results with *p* ≤ 0.05 were deemed statistically significant. Asterisk (*∗*) indicates the statistically significant differences.

LPS treatment suppressed (*p* < 0.05) GHR protein expression in the liver both 3 and 9 h after the treatment compared to this receptor expression in the control animals (Figure 2; panel A). Moreover, the administration of LPS reduced (*p* < 0.05) the protein level of IGF1 in the liver 3 h after the treatment. On the other hand, in the animals sacrificed 3h after the endotoxin administration the IGF1 expression in the liver was at the same level in comparison to the control group (Figure 2; panel B)

### 3.3. Hypothalamus

It was found that LPS treatment reduced (*p* < 0.05) GHRH gene expression in the MBH of ewes euthanized 9 h after the treatment. On the other hand, the expression of the gene encoding GHRH in the group sacrificed 3 h after the LPS treatment did not differ from this gene expression in the control group (Table 2). In the DMH, the administration of LPS caused an increase (*p* < 0.05) SST gene expression in the animals euthanized 3h after the treatment, while in the group sacrificed 9 h after the LPS injection, no changes in this gene expression were stated (Table 2).

### 3.4. Anterior Pituitary

It was found that the expression of gene encoding GHRHR was increased (*p* < 0.05) in the AP collected 3 h after the LPS treatment, while no changes in this gene expression were stated in the AP collected 9 h after the LPS injection. It was also determined that the LPS injection decreased (*p* < 0.05) the expression of genes encoding SSTR1, SSTR2, SSTR3, and SSTR5 in the AP in animals euthanized 3 h and 9 h after the endotoxin administration. It was stated that the expression of gene encoding GH increased (*p* < 0.05) in the AP from ewes sacrificed 3 h after the LPS injection, on the other hand, this effect of endotoxin treatment was not observed in the glands collected 9 h after the injection (Table 3).

### 3.5. Liver

It was stated that the LPS-induced inflammation suppressed (*p* < 0.05) expression of GHR gene in the liver collected 3 h and 9 h after the endotoxin administration in comparison to the control groups. The expression of IGF1 mRNA was reduced (*p* < 0.05) by LPS treatment in the liver but only in tissues collected 9 h after the injection. It was also found that the expression of gene encoding STAT5B in the liver was decreased (*p* < 0.05) after the LPS treatment in all LPS-treated groups. The gene expression of JAK2 was stimulated (*p* < 0.05) by the LPS injection but only in the liver dissected from 3 h after the treatment. Whereas the expression of suppressor of cytokine signaling 3 (SOCS3) mRNA was increased (*p* < 0.05) in both LPS-treated groups. Endotoxin-induced inflammation reduced (*p* < 0.05) SIRT1 mRNA expression in the liver, but only in those collected 3h after the treatment. Moreover, it was also found that the expression of gene encoding FGF21 was increased (*p* < 0.05) only in the livers collected 9 h after the injection of LPS (Table 4).

## 4. Discussion

Our study showed that the inflammation caused by the LPS treatment stimulates the release of GH and the expression of its encoding gene in the AP in the 3 h after the injection. On the other hand, this stimulatory effect of acute LPS administration on the GH release seems to expire over time because in the 9 h after the LPS administration, this stimulatory effect on the GH gene expression has not been observed, while the circulating level of GH was still elevated. The lack of parallelism in the changes in the blood concentration of GH and GH gene expression in the pituitary may result partly from the nature of this hormone's secretion. In the pituitary gland, GH is not released immediately after synthesis in the AP. Following synthesis by polysomes attached to the endoplasmic reticulum, the GH hormone is packaged in the Golgi apparatus, forming secretory granules that are then sent to the plasma membrane and stored until stimulation [43–50]. Moreover, the reason for still elevated circulating GH concentration at 9 h after the LPS treatment may be an intense release of this hormone in the earlier period; however, it should be mentioned that GH is characterized by a relatively short half-life. In the organism, GH is cleared via the kidneys and/or GHR internalization and has a half-life of approximately 15–20 min [51]. It is worth mentioning that the stimulatory effect of acute LPS administration on GH release has been previously reported both in sheep [52] and humans [53]. In another study, acute endotoxin administration in rats decreased the concentration of circulating GH [54]. However, a more recent study suggested a relationship between the dose of the toxin and the direction of its effect on GH secretion. It was shown that the LPS administrated at low doses stimulated GH release but at high doses, it suppressed this hormone secretion [55].

Interestingly, our study on the sheep model suggests that the mechanisms leading to the inflammatory-dependent stimulation of GH secretion are not entirely clear. The secretion of GH in the AP is regulated by the hypothalamus and major regulatory factors include GHRH and SST [56, 57]. Our results showed that LPS did not increase GHRH gene expression in the MBH and even the level of GHRH mRNA was reduced 9 h after the endotoxin administration. Acute stress induced by LPS injection did not reduce SST mRNA expression in the DMH and even stimulated this gene expression in the 3 h after the treatment. These all suggest that in ewes, acute inflammation did not stimulate GH secretion via modulation of its hypothalamic modulators. The results of our study indicate that inflammation induced by acute LPS injection modulates GH secretion acting primarily at the pituitary level. This effect could be included directly by the circulating endotoxin because our previous study showed the gene expression of *TLR4* directly in the AP [58]. Moreover, the *in vitro* study demonstrated that endotoxin directly stimulated GH release from cultured ovine pituitary cells [52]. However, at least partially the inflammatory-dependent changes in the GH secretion in the AP may be caused by blood-borne as well as locally synthetized inflammatory cytokines. Our recent study showed that endotoxin injection induced time-dependent changes in the gene expression of proinflammatory cytokines and their corresponding receptors [59]. This study showed that at the early stage of inflammatory response, the expression of *IL1B*, *IL6,* and *TNFA* was increased in the AP. This stimulatory effect of inflammation on the gene expression of these cytokines gradually disappeared and at the end of the experiment, 9 hours after LPS injection, only an increase in the gene expression of *IL6* was determined. These three cytokines can modulate GH secretion in the pituitary cells. The role of IL1B in the direct regulation of GH secretion remains ambiguous. The *in vitro* experiment on porcine pituitary cells showed that IL1B increased GH output but reduced the galanin-induced GH secretion [60]. The study on rat anterior pituitary cells also showed stimulatory direct action of IL1B on GH secretion [61]. On the other hand, the study on rat pituitary cells under serum-free conditions suggested a generally inhibitory action of IL1B on GH release [62]. The GH secretion in the AP could be modulated also by TNFA; *in vitro* experiments showed that this cytokine decreased GRH-stimulated GH release from cultured ovine pituitary cells [63]. It is worth mentioning that our previous study showed that acute LPS injection-stimulated *TNFA* gene expression has the shortest reaction time among the studied cytokines [59]. The inflammatory cytokine whose gene expression remained elevated in the AP throughout the entire experiment was IL6. Previous *in vitro* studies showed that IL6 stimulated GH synthesis and release [64, 65]. The results of an *in vitro* study on pituitary cells from adult pigs showed that the role of IL6 in the modulation of GH secretion could be even more significant because it was found that IL6 not only stimulated GH release but also potentiated the effect of GH releasers [60]. It is worth mentioning that the stimulatory effect of IL6 on the release of GH was also reported in the study on men infused with recombinant human (rh) IL6 via an antecubital vein. This showed that IL6 infusion led to a significant increase in GH, peaking 1 h after the beginning of the infusion [66]. Moreover, research conducted on humans has shown a dose-dependent effect of the stimulatory influence of IL6 on the GH secretion and the highest peak was reached when a dose of 3 *μ*g/kg of body weight was used [67]. On the other side, in the same study, no effect of TNFA on the basal secretion of GH was stated [67]. However, TNFA and IL1B are recognized as the most potent inducers of IL6 encoding gene expression and it has been firmly established that nuclear factor kappa-light-chain-enhancer of activated B cells (NF-*κ*B) plays a pivotal role in orchestrating this regulatory process [68, 69]. Therefore, both IL1B and TNFA may also exert an indirect effect on GH through the stimulation of IL6.

Interestingly, although inflammation had no stimulatory effect on the transcription of GHRH in the hypothalamus, in the pituitaries collected 3 h after LPS administration, an increased GHRHR mRNA level was stated. GHRHR plays a pivotal role in the regulation of GH synthesis and secretion because it is responsible for the signal transduction of the hypothalamic GHRH. GHRH stimulates GH secretion from somatotropic cells of the AP via a pathway that involves GHRH receptor activation of adenylyl cyclase and increased cyclic adenosine monophosphate (cAMP) production [70]. Increased gene expression of *GHRHR* in the AP suggests increased sensitivity of this gland in the first hours after LPS administration to GHRH stimulation, which at least partially might influence on stimulation of GH secretion during acute immune/inflammatory challenges. It is worth mentioning that a transient increase in the gene expression of *GHRH* in the AP may be caused by stress induced by inflammation. It is well known that LPS injection activates the hypothalamic-pituitary-adrenal axis leading to an increase in the blood level of corticosteroids such as cortisol [71] and corticosterone [72]. *In vitro* study on the rat somatotroph cell line, MtT/S showed that corticosterone stimulated the expression of *GHRHR* [73]. Our results also showed that in most cases, the expression of SST receptors was reduced in the AP collected from endotoxin-treated ewes. SST is considered to be one of the main inhibitors of the HPS axis and a suppressor of GH secretion. It was previously reported that SST inhibited GH secretion by reducing intracellular cAMP and/or hyperpolarizing the cells through SST receptors and it is involved in the transcriptional regulation of the GH gene [73]. Therefore, inflammatory-dependent changes in the pituitary expression of receptors for hypothalamic GHRH and SST may at least partially result in increased GH secretion.

Our study showed that systemic inflammation caused by the LPS administration suppresses the IGF1 gene and protein expression in the liver in 9 h after the treatment. Interestingly, this increase was found despite the elevated circulating concentration of GH, which is considered to be a potent stimulator of IGF1 production [74, 75]. However, we found that the same as in the case of the AP, the liver endotoxin injection caused a significant reduction of GHR mRNA expression. This result is consistent with the results of a previous *in vitro* study which showed that LPS treatment through both MyD88-dependent and -independent TLR4 signaling pathways inhibited GHR promoter activity leading to the inhibition of *GHR* gene expression [76]. Our results establish a novel cytokine-independent mechanism for a decrease in *GHR* expression in bacterial sepsis. This may suggest reduced expression of *GHR* in the liver which in turn causes decreased sensitivity of this organ to GH stimulation, a state that can be described as resistance to GH action. This may explain why increased GH release did not induce an increase in IGF1 secretion. However, it is worth pointing out that inflammation may also cause disturbances in GHR signal transduction. It was found that endotoxin treatment decreased the expression of the gene encoding STAT5B and at the same time increased SOCS3 mRNA expression. This indicates that inflammation induces a postreceptor inhibitory mechanism. It is well known that GH regulates IGF1 production through activation of STAT5B signaling cascade and that STAT5B is required for GH-induced IGF1 mRNA expression in the liver [77, 78]. Therefore, inflammatory-dependent suppression of *STAT5B* expression in the liver may be another mechanism involved in the inhibition of IGF1 secretion. On the other hand, it was found that inflammation increased gene expression of *SOCS3* in the ovine liver, which also may profoundly negatively influence the GHR-JAK2-STAT transduction pathway. SOCS3 exerts inhibitory control over the GH-mediated JAK-STAT signaling pathway through various mechanisms. One mode of action involves SOCS3 competitively impeding the phosphorylation of STAT5B, a downstream effector in the JAK-STAT cascade. In addition, SOCS3 can directly bind to the GHR, hindering the recruitment and phosphorylation of STAT5B [79, 80]. Moreover, SOCS3's interaction with GHR initiates the formation of a complex, leading to the degradation of the GHR-JAK2 complex through processes like ubiquitination. The presence of the SOCS box in SOCS3 facilitates the recruitment of Elongin BC, a complex involved in the ubiquitin-proteasome system. This interaction contributes to the ubiquitination and subsequent degradation of GHR and JAK2, culminating in the attenuation of JAK2 activity [81, 82]. Therefore, the inflammatory-dependent inhibition of IGF1 secretion could result largely from increased *SOCS3* expression. Increased expression of *SOCS3* may also explain why the increase in the *JAK2* gene expression determined in the livers collected 3 h after LPS injection did not influence IGF1 production. Our study suggests that another inhibitor of GH signaling SIRT1 seems to be not involved in the suppression of GHR transduction during acute inflammation. It was found that endotoxin injection even decreased the gene expression of *SIRT1* in the livers of ewes 3 h after the treatment, whereas 9 h after the treatment SIRT1 mRNA expression did not differ from the control. It should be mentioned that SIRT1 due to its multidirectional inhibitory action directed at the GHR signal transduction is considered to be involved in the pathophysiology of GHres [26]. Our results suggest that in the GHres induced by acute immune stress, the role of SIRT1 is marginal. In contrast, we found increased gene expression of *FGF21* in the liver collected 9h after LPS injection. This allows us to assume that FGF21 could be also involved in the induction of GHres at the later stages of the inflammatory response. FGF21 can act as an endocrine as well as a paracrine factor and is considered to be an important negative regulator of mammalian growth [83]. It was found that in the liver, FGF21 lowers the concentrations of the active form of STAT5B, a major mediator of GH actions, and causes corresponding decreases in the expression of its target genes including *IGF1*. FGF21 also induces hepatic expression of *IGF1* binding protein 1 and suppressor of cytokine signaling 2, which blunt GH signaling [24]. It is worth mentioning that increased expression of *FGF21* gene during endotoxin-induced inflammation may result from its anti-inflammatory properties which were reported in both in vitro and in vivo studies [84, 85]. It was found that its administration has a protective effect from the toxicity of LPS and sepsis [86].

## 5. Conclusions

Our study on the sheep model showed that inflammation disturbs the activity of the somatotropic axis on the one hand stimulating the secretion of GH on the other hand reducing the sensitivity of the liver to this hormone action via direct reduction of *GHR* expression as well as by the activation of mechanism inhibiting the GHR signal transduction pathway. The effect of GHres in the liver was suppressed IGF1 secretion which in the long term may have negative consequences for growth and development. It seems that inflammation-induced resistance to GH may be one of the important elements through which inflammation negatively affects the body's condition. Because the sheep is a recognized animal model in immunology and neuroendocrinology research, better understanding of the processes leading to the development of GHres as well as the consequences of GHres for growth and development may be valuable for human medicine.

## Figures and Tables

**Table 1:** Primer descriptions.

Gene symbol	Primer		Reference
	Forward	Reverse	
GAPDH	TGACCCCTTCATTGACCTTC	GATCTCGCTCCTGGAAGATG	[38]
HDAC1	CTGGGGACCTACGGGATATT	GACATGACCGGCTTGAAAAT	[39]
PPIC	TGGCACTGGTGGTATAAGCA	GGGCTTGGTCAAGGTGATAA	[38]
B2M	CTTCTGTCCCACGCTGAGTT	GGTGCTTAGAGGTCTCG	[40]
GHRH	CCTCTCAGGATTCCACGGTA	CGTACCTTTGCTCCTTGCTC	[41]
GHRHR	CTTCTCTCACTTCAGCTTGG	GGATTTCTCCTTCAGTCAGC	[41]
SSTR1	ACTCCATGGTCATCTACGTG	GAAGCAATGTGGAGGTGAC	[41]
SSTR2	TCTCTCTGCTGGTCATCTTG	CGTAGATGATGAACCCTGTG	[41]
SSTR3	CACTGGTCTATCTGGTGGTG	TTGAGGATGTAGACATTGGTG	[41]
SSTR5	TGGTCATCTATGTGGTCCTG	AGTAGGAGATGGCGTTTTG	[41]
GH	TTCCTCAGCAGAGTCTTCACC	GGGGTAACATCTTCCAGCTC	[41]
GHR	ACTGTTAGCCCAAGTATTCC	ATATGGCAAGTTCAGTGAGG	[41]
STAT5B	ATTACACCCCAGTTCCATGC	AGAGGCGCTCACAAACTCAG	Originally designed
JAK2	ATTCAGAGTCTTTCTTTGAAGC	AATATTCTCCTCTCCACAGACAC	Originally designed
SOCS3	CCAGGAGAGCCTATTACATT	GTCTTCCGACAGAGATGTTG	Originally designed
IGF-1	ATCGTGGATGAGTGCTGCTT	ATGTACTTCCTTCTGAGCCTTGG	Originally designed
SIRT1	AAGGAAAACTACTTCGCAAC	TCCTCGTACAGCTTCACAGT	Originally designed
FGF21	CAGAGTCCCGAAAGTCTCTT	AAAGTGCAGCGATCCATAC	Originally designed

**Table 2:** The effect of lipopolysaccharide treatment on the growth hormone-releasing hormone (GHRH) and somatostatin (SST) genes in the mediobasal and dorsomedial hypothalamic nuclei.

Gene	Group	Time groups		Tissue
		3 h	9 h	
GHRH	Control	1 ± 0.05	1 ± 0.07	MBH
	*p* value	0.11	0.008	
	LPS	0.89 ± 0.03^−^	0.75 ± 0.02^↓^	

SST	Control	1 ± 0.05	1 ± 0.12	DMH
	*p* value	0.017	0.82	
	LPS	1.32^↑^	1.04^−^	

The results are presented in arbitrary units as the mean ± standard error of the mean (SEM) and results with *p* ≤ 0.05 were deemed statistically significant. Arrows indicate significant differences between the groups, while dashes indicate no effect.

**Table 3:** The effect of lipopolysaccharide injection on the relative expression of the following genes: growth hormone-releasing hormone receptor (GHRH), somatostatin receptors (SSTR) 1–3 and 5, and growth hormone (GH) at the anterior pituitary level.

Gene	Group	Time groups	
		3 h	9 h
GHRHR	Control	1 ± 0.14	1 ± 0.16
	*p* value	0.02	0.67
	LPS	1.60 ± 0.15^↑^	1.13 ± 0.20^−^

SSTR1	Control	1 ± 0.07	1 ± 0.14
	*p* value	0.003	0.02
	LPS	0.48 ± 0.10^↓^	0.59 ± 0.03^↓^

SSTR2	Control	1 ± 0.06	1 ± 0.10
	*p* value	0.000035	0.003
	LPS	0.44 ± 0.05^↓^	0.57 ± 0.03^↓^

SSTR3	Control	1 ± 0.07	1 ± 0.04
	*p* value	0.0002	0.0000088
	LPS	0.50 ± 0.04^↓^	0.51 ± 0.03^↓^

SSTR5	Control	1 ± 0.10	1 ± 0.04
	*p* value	0.00005	0.0006
	LPS	0.26 ± 0.01^↓^	0.72 ± 0.04^↓^

GH	Control	1 ± 0.15	1 ± 0.13
	*p* value	0.001	0.99
	LPS	1.80 ± 0.06^↑^	1.00 ± 0.10^−^

The results are presented in arbitrary units as the mean ± standard error of the mean (SEM) and results with *p* ≤ 0.05 were deemed statistically significant. Arrows indicate significant differences between the groups, while dashes indicate no effect.

**Table 4:** The effect of lipopolysaccharide injection on the relative expression of the following genes: growth hormone receptor (GHR), insulin-like growth factor 1 (IGF1), signal transducer and activator of transcription 5B (STAT5B), janus kinase 2 (JAK2), suppressor of cytokine signaling 3 (SOCS3), sirtuin 1 (SIRT1), and fibroblast growth factor 21 (FGF21) at the liver level.

Gene	Group	Time groups	
		3 h	9 h
GHR	Control	1 ± 0.08	1 ± 0.10
	*p* value	0.0003	0.008
	LPS	0.47 ± 0.05^↓^	0.58 ± 0.06^↓^

IGF1	Control	1 ± 0.19	1 ± 0.12
	*p* value	0.46	0.03
	LPS	0.87 ± 0.15^−^	0.67 ± 0.08^↓^

STAT5B	Control	1 ± 0.11	1 ± 0.09
	*p* value	0.0007	0.04
	LPS	0.41 ± 0.03^↓^	0.18 ± 0.08^↓^

JAK2	Control	1 ± 0.08	1 ± 0.10
	*p* value	0.0005	0.70
	LPS	2.16 ± 0.20^↑^	1.08 ± 0.14^−^

SOCS3	Control	1 ± 0.16	1 ± 0.16
	*p* value	0.006	0.00007
	LPS	1.99 ± 0.21^↑^	3.30 ± 0.28^↑^

SIRT1	Control	1 ± 0.03	1 ± 0.11
	*p* value	0.0009	0.07
	LPS	0.61 ± 0.07^↓^	1.29 ± 0.12^−^

FGF21	Control	1 ± 0.07	1 ± 0.19
	*p* value	0.07	0.003
	LPS	0.81 ± 0.05^−^	2.74 ± 0.37^↑^

The results are presented in arbitrary units as the mean ± standard error of the mean (SEM) and results with *p* ≤ 0.05 were deemed statistically significant. Arrows indicate significant differences between the groups, while dashes indicate no effect.

## Data Availability

The data used to support the findings of this study are included within the article.

## References

[1] Barabutis N., Akhter M. S., Kubra K.-T., Jackson K. (2022). Growth hormone–releasing hormone in endothelial inflammation. *Endocrinology*.

[2] Herman A., Romanowicz K., Tomaszewska-Zaremba D. (2010). Effect of LPS on reproductive system at the level of the pituitary of anestrous ewes: effect of LPS on reproductive system at the pituitary level. *Reproduction in Domestic Animals*.

[3] Zhao Y., Xiao X., Frank S. J., Lin H. Y., Xia Y. (2014). Distinct mechanisms of induction of hepatic growth hormone resistance by endogenous IL-6, TNF-*α*, and IL-1*β*. *American Journal of Physiology-Endocrinology and Metabolism*.

[4] Haddad J. J., Saadé N. E., Safieh-Garabedian B. (2002). Cytokines and neuro-immune-endocrine interactions: a role for the hypothalamic-pituitary-adrenal revolving Axis. *Journal of Neuroimmunology*.

[5] Baumann G. (1994). Growth hormone-binding proteins: state of the art. *Journal of Endocrinology*.

[6] Colson A., Willems B., Thissen J. (2003). Inhibition of TNF-alpha production by pentoxifylline does not prevent endotoxin-induced decrease in serum IGF-I. *Journal of Endocrinology*.

[7] Briard N., Dadoun F., Pommier G. (2000). IGF-I/IGFBPs system response to endotoxin challenge in sheep. *Journal of Endocrinology*.

[8] Daniel J. A., Elsasser T. H., Martínez A., Steele B., Whitlock B. K., Sartin J. L. (2005). Interleukin-1*β* and tumor necrosis factor-*α* mediation of endotoxin action on growth hormone. *American Journal of Physiology-Endocrinology and Metabolism*.

[9] Salomon F., Cuneo R. C., Hesp R., Sönksen P. H. (1989). The effects of treatment with recombinant human growth hormone on body composition and metabolism in adults with growth hormone deficiency. *New England Journal of Medicine*.

[10] Binnerts A., Swart G. R., Wilson J. H. (1992). The effect of growth hormone administration in growth hormone deficient adults on bone, protein, carbohydrate and lipid homeostasis, as well as on body composition. *Clinical Endocrinology*.

[11] Wüster C., Slenczka E., Ziegler R. (1991). Increased prevalence of osteoporosis and arteriosclerosis in conventionally substituted anterior pituitary insufficiency: need for additional growth hormone substitution?. *Klinische Wochenschrift*.

[12] Cuneo R. C., Salomon F., Wiles M., Sönksen P. (1990). Skeletal muscle performance in adults with growth hormone deficiency. *Hormone Research*.

[13] Rosén T., Bengtsson B.-Å. (1990). Premature mortality due to cardiovascular disease in hypopituitarism. *The Lancet*.

[14] Chong P. K. K., Jung R. T., Scrimgeour C. M., Rennie M. J., Paterson C. R. (1994). Energy expenditure and body composition in growth hormone deficient adults on exogenous growth hormone. *Clinical Endocrinology*.

[15] Carroll P. V., Christ the members of Growth Hormon E. R., Bengtsson B. Å. (1998). Growth hormone deficiency in adulthood and the effects of growth hormone replacement: a review. *The Journal of Clinical Endocrinology & Metabolism*.

[16] Attanasio A. F., Lamberts S. W. J., Matranga A. M. C. (1997). Adult growth hormone (GH)-Deficient patients demonstrate heterogeneity between childhood onset and adult onset before and during human GH treatment^1^. *The Journal of Clinical Endocrinology & Metabolism*.

[17] Cuneo R. C., Salomon F., Watts G. F., Hesp R., Sönksen P. H. (1993). Growth hormone treatment improves serum lipids and lipoproteins in adults with growth hormone deficiency. *Metabolism*.

[18] Zaczek D., Hammond J., Suen L. (2002). Impact of growth hormone resistance on female reproductive function: new insights from growth hormone receptor knockout Mice1. *Biology of Reproduction*.

[19] Guevara-Aguirre J., Rosenbloom A. L. (2015). Obesity, diabetes and cancer: insight into the relationship from a cohort with growth hormone receptor deficiency. *Diabetologia*.

[20] Fisher F. M., Maratos-Flier E. (2016). Understanding the physiology of FGF21. *Annual Review of Physiology*.

[21] Nishimura T., Nakatake Y., Konishi M., Itoh N. (2000). Identification of a novel FGF, FGF-21, preferentially expressed in the liver. *Biochimica et Biophysica Acta (BBA)-Gene Structure and Expression*.

[22] Zhang X., Yeung D. C. Y., Karpisek M. (2008). Serum FGF21 levels are increased in obesity and are independently associated with the metabolic syndrome in humans. *Diabetes*.

[23] Fazeli P. K., Misra M., Goldstein M., Miller K. K., Klibanski A. (2010). Fibroblast growth factor-21 may mediate growth hormone resistance in anorexia nervosa. *The Journal of Clinical Endocrinology & Metabolism*.

[24] Inagaki T., Lin V. Y., Goetz R., Mohammadi M., Mangelsdorf D. J., Kliewer S. A. (2008). Inhibition of growth hormone signaling by the fasting-induced hormone FGF21. *Cell Metabolism*.

[25] Brooks A. J., Waters M. J. (2010). The growth hormone receptor: mechanism of activation and clinical implications. *Nature Reviews Endocrinology*.

[26] Wójcik M., Krawczyńska A., Antushevich H., Herman A. (2018). Post-receptor inhibitors of the GHR-JAK2-STAT pathway in the growth hormone signal transduction. *IJMS*.

[27] Kimura A., Naka T., Muta T. (2005). Suppressor of cytokine signaling-1 selectively inhibits LPS-induced IL-6 production by regulating JAK–STAT. *Proceedings of the National Academy of Sciences of the United States of America*.

[28] Yu Y., Li S., Liu Y. (2015). Fibroblast growth factor 21 (FGF21) ameliorates collagen-induced arthritis through modulating oxidative stress and suppressing nuclear factor-kappa B pathway. *International Immunopharmacology*.

[29] Li Y., Wong K., Giles A. (2014). Hepatic SIRT1 attenuates hepatic steatosis and controls energy balance in mice by inducing fibroblast growth factor 21. *Gastroenterology*.

[30] Planavila A., Redondo-Angulo I., Ribas F. (2015). Fibroblast growth factor 21 protects the heart from oxidative stress. *Cardiovascular Research*.

[31] Blander G., Guarente L. (2004). The Sir2 family of protein deacetylases. *Annual Review of Biochemistry*.

[32] Haigis M. C., Sinclair D. A. (2010). Mammalian sirtuins: biological insights and disease relevance. *Annual Review of Pathology: Mechanisms of Disease*.

[33] Chau M. D. L., Gao J., Yang Q., Wu Z., Gromada J. (2010). Fibroblast growth factor 21 regulates energy metabolism by activating the AMPK–SIRT1–PGC-1*α* pathway. *Proceedings of the National Academy of Sciences of the United States of America*.

[34] Yamamoto M., Iguchi G., Fukuoka H. (2013). SIRT1 regulates adaptive response of the growth hormone--insulin-like growth factor-I Axis under fasting conditions in liver. *Proceedings of the National Academy of Sciences of the United States of America*.

[35] Udy G. B., Towers R. P., Snell R. G. (1997). Requirement of STAT5b for sexual dimorphism of body growth rates and liver gene expression. *Proceedings of the National Academy of Sciences*.

[36] Liu T. F., McCall C. E. (2013). Deacetylation by SIRT1 reprograms inflammation and cancer. *Genes & Cancer*.

[37] Przybył B., Wójcik-Gładysz A., Gajewska A., Szlis M. (2021). Brain-derived neurotrophic factor (BDNF) affects somatotrophicaxis activity in sheep. *Journal of Animal and Feed Sciences*.

[38] Haziak K., Herman A. P., Tomaszewska-Zaremba D. (2014). Effects of central injection of anti-LPS antibody and blockade of TLR4 on GnRH/LH secretion during immunological stress in anestrous ewes. *Mediators of Inflammation*.

[39] Szczepkowska A., Bochenek J., Wójcik M. (2022). Effect of caffeine on adenosine and ryanodine receptorgene expression in the hypothalamus, pituitary, and choroidplexus in ewes under basal and LPS challenge conditions. *Journal of Animal and Feed Sciences*.

[40] Wojtulewicz K., Tomczyk M., Wójcik M. (2023). Circadian and seasonal changesin the expression of clock genes in the ovine pars tuberalis. *Journal of Animal and Feed Sciences*.

[41] Wójcik M., Krawczyńska A., Zieba D. A., Antushevich H., Herman A. P. (2023). Influence of leptin on the secretion of growth hormone in ewes under different photoperiodic conditions. *IJMS*.

[42] Dvorak P., Becka S., Krejci P., Chrpova M. (1978). Radioimmunoassay of bovine growth hormone. *Radiochemical & Radioanalytical Letters*.

[43] Palade G. E. (1955). A small particulate component of the cytoplasm. *The Journal of Cell Biology*.

[44] Palade G. (1975). Intracellular aspects of the process of protein synthesis. *Science*.

[45] Farquhar M. G., Wellings S. R. (1957). Electron microscopic evidence suggesting secretory granule formation within the Golgi apparatus. *The Journal of Cell Biology*.

[46] Caro L. G., Palade G. E. (1964). Protein synthesis, storage, and discharge in the pancreatic exocrine cell. *The Journal of Cell Biology*.

[47] Jamieson J. D., Palade G. E. (1966). Role of the Golgi complex in the intracellular transport of secretory proteins. *Proceedings of the National Academy of Sciences of the United States of America*.

[48] Jamieson J. D., Palade G. E. (1967). Intracellular transport of secretory proteins in the pancreatic exocrine cell. I. Role of the peripheral elements of the Golgi complex. *The Journal of Cell Biology*.

[49] Jamieson J. D., Palade G. E. (1967). Intracellular transport of secretory proteins in the pancreatic exocrine cell. *The Journal of Cell Biology*.

[50] Florea A., El Hof F. A., Hazi G. M., Oprea M. C. (2019). Bee venom stimulates hormone secretion in rat somatotroph and corticotroph cells: digital image analysis of secretory granules. *Microscopy and Microanalysis*.

[51] Van Der Lely A. J., Kopchick J. J. (2006). Growth hormone receptor antagonists. *Neuroendocrinology*.

[52] Daniel J. A., Whitlock B. K., Wagner C. G., Sartin J. L. (2002). Regulation of the growth hormone and luteinizing hormone response to endotoxin in sheep. *Domestic Animal Endocrinology*.

[53] Lang C. H., Pollard V., Fan J. (1997). Acute alterations in growth hormone-insulin-like growth factor Axis in humans injected with endotoxin. *American Journal of Physiology - Regulatory, Integrative and Comparative Physiology*.

[54] Soto L., Martin A., Millan S., Vara E., Lopez-Calderon A. (1998). Effects of endotoxin lipopolysaccharide administration on the somatotropic Axis. *Journal of Endocrinology*.

[55] Priego T., Granado M., Ibanez De Caceres I., Martin A., Villanua M., Lopez-Calderon A. (2003). Endotoxin at low doses stimulates pituitary GH whereas it decreases IGF-I and IGF-binding protein-3 in rats. *Journal of Endocrinology*.

[56] Müller E. E., Locatelli V., Cocchi D. (1999). Neuroendocrine control of growth hormone secretion. *Physiological Reviews*.

[57] Kato Y., Murakami Y., Sohmiya M., Nishiki M. (2002). Regulation of human growth hormone secretion and its disorders. *Internal Medicine*.

[58] Haziak K., Herman A., Tomaszewska-Zaremba D. (2013). The effect of LPS on LH release and gene expression of *LH-β, GnRH-R* and *TLR4* in the anterior pituitary of follicular phase ewes – an *in vitro* study. *Journal of Animal and Feed Sciences*.

[59] Wójcik M., Zięba D. A., Tomczyk M. (2023). Time-dependent effect of inflammation on the gene expressionof pro-inflammatory cytokines and their receptors at the differentlevels of the somatotropic Axis in Ewe. *Journal of Animal and Feed Sciences*.

[60] Mainardi G. L., Saleri R., Tamanini C., Baratta M. (2002). Effects of interleukin-1-beta, interleukin-6 and tumor necrosis factor-alpha, alone or in association with hexarelin or galanin, on growth hormone gene expression and growth hormone release from pig pituitary cells. *Hormone Research in Paediatrícs*.

[61] Niimi M., Sato M., Wada Y., Tamaki M., Takahara J., Kawanishi K. (1994). Analysis of growth hormone release from rat anterior pituitary cells by reverse hemolytic plaque assay: influence of interleukin-1. *Life Sciences*.

[62] Renner U., Newton C. J., Pagotto U., Sauer J., Arzt E., Stalla G. K. (1995). Involvement of interleukin-1 and interleukin-1 receptor antagonist in rat pituitary cell growth regulation. *Endocrinology*.

[63] Fry C. L., Gunter D. R., McMahon C. D., Steele B., Sartin J. L. (1998). Cytokine-mediated growth hormone release from cultured ovine pituitary cells. *Neuroendocrinology*.

[64] Spangelo B. L., Judd A. M., Isakson P. C., Macleod R. M. (1989). INTERLEUKIN-6 stimulates anterior pituitary hormone release *in vitro*. *Endocrinology*.

[65] Gong F., Shi Y., Deng J. (2006). The regulatory mechanism by which interleukin-6 stimulates GH-gene expression in rat GH3 cells. *Journal of Endocrinology*.

[66] Nemet D., Eliakim A., Zaldivar F., Cooper D. M. (2006). Effect of rhIL-6 infusion on GH⟶IGF-I Axis mediators in humans. *American Journal of Physiology - Regulatory, Integrative and Comparative Physiology*.

[67] Tsigos C., Papanicolaou D. A., Defensor R., Mitsiadis C. S., Kyrou I., Chrousos G. P. (1997). Dose effects of recombinant human lnterleukin-6 on pituitary hormone secretion and energy expenditure. *Neuroendocrinology*.

[68] Merola M., Blanchard B., Tovey M. G. (1996). The *κ*B enhancer of the human interleukin-6 promoter is necessary and sufficient to confer an IL-1*β* and TNF-*α* response in transfected human cell lines: requirement for members of the C/EBP family for activity. *Journal of Interferon and Cytokine Research*.

[69] Furia A., Confalone E., D’Alessio G. (2010). IL-6 induction by TNF*α* and IL-1*β* in an osteoblast-like cell line. *International Journal of Biomedical Sciences*.

[70] Cunha S. R., Mayo K. E. (2002). Ghrelin and growth hormone (GH) secretagogues potentiate GH-releasing hormone (GHRH)-Induced cyclic adenosine 3′,5′-monophosphate production in cells expressing transfected GHRH and GH secretagogue receptors. *Endocrinology*.

[71] Herman A. P., Krawczyńska A., Bochenek J. (2013). The effect of rivastigmine on the LPS-induced suppression of GnRH/LH secretion during the follicular phase of the estrous cycle in ewes. *Animal Reproduction Science*.

[72] Gross J. J., Schwinn A.-C., Bruckmaier R. M. (2021). Free and bound cortisol, corticosterone, and metabolic adaptations during the early inflammatory response to an intramammary lipopolysaccharide challenge in dairy cows. *Domestic Animal Endocrinology*.

[73] Morishita M., Iwasaki Y., Onishi A. (2003). The effects of GH-releasing hormone/somatostatin on the 5’-promoter activity of the GH gene in vitro. *Journal of Molecular Endocrinology*.

[74] Gardner D. G., Greenspan F. S. (2011). *Greenspan’s Basic and Clinical Endocrinology*.

[75] Szcześniak P., Dudarewicz M., Michalak Ł., Orszulak-Michalak D. (2019). Insulin-like growth factor I (IGF-I) and its present significance in digestive system diseases. *Postepy Higieny I Medycyny Doswladczalnej*.

[76] Dejkhamron P., Thimmarayappa J., Kotlyarevska K. (2007). Lipopolysaccharide (LPS) directly suppresses growth hormone receptor (GHR) expression through MyD88-dependent and -independent toll-like receptor-4/MD2 complex signaling pathways. *Molecular and Cellular Endocrinology*.

[77] Davey H. W., Xie T., McLachlan M. J., Wilkins R. J., Waxman D. J., Grattan D. R. (2001). STAT5b is required for GH-induced liver igf-I gene expression. *Endocrinology*.

[78] Hwa V. (2016). STAT5B deficiency: impacts on human growth and immunity. *Growth Hormone & IGF Research*.

[79] Sasaki A., Yasukawa H., Suzuki A. (1999). Cytokine‐inducible SH2 protein‐3 (CIS3/SOCS3) inhibits *janus* tyrosine kinase by binding through the N‐terminal kinase inhibitory region as well as SH2 domain. *Genes to Cells*.

[80] Yasukawa H. (1999). The JAK-binding protein JAB inhibits janus tyrosine kinase activity through binding in the activation loop. *The EMBO Journal*.

[81] Babon J. J., Kershaw N. J., Murphy J. M. (2012). Suppression of cytokine signaling by SOCS3: characterization of the mode of inhibition and the basis of its specificity. *Immunity*.

[82] Hansen J. A., Lindberg K., Hilton D. J., Nielsen J. H., Billestrup N. (1999). Mechanism of inhibition of growth hormone receptor signaling by suppressor of cytokine signaling proteins. *Molecular Endocrinology*.

[83] Grunwald T., Luca F. (2015). Role of fibroblast growth factor 21 (FGF21) in the regulation of statural growth. *Contemporary Perspectives in Rehabilitation*.

[84] Wang N., Zhao T., Li S. (2019). Fibroblast growth factor 21 exerts its anti‐inflammatory effects on multiple cell types of adipose tissue in obesity. *Obesity*.

[85] Li J., Gong L., Zhang R. (2021). Fibroblast growth factor 21 inhibited inflammation and fibrosis after myocardial infarction via EGR1. *European Journal of Pharmacology*.

[86] Ogawa Y., Kurosu H., Yamamoto M. (2007). *β*Klotho is required for metabolic activity of fibroblast growth factor 21. *Proceedings of the National Academy of Sciences of the United States of America*.

